# Supplementary material for: Tead1a Initiates Transcriptional Priming Through the TEAD1a/YAP‐Notch1‐Spi1/Cebpα Axis to Promote Neutrophil Fate
Source: Adv Sci (Weinh). 2025 Aug 28;12(41):e05441. doi: 10.1002/advs.202505441 (PMC12591127; doi:10.1002/advs.202505441)
Supplement: Supplementary file 4 — Supplementary Dataset [file ADVS-12-e05441-s004.zip › 3-2.zip!/3-2/02-2.cluster/cluster4/Enrich/ego_BP.pdf]

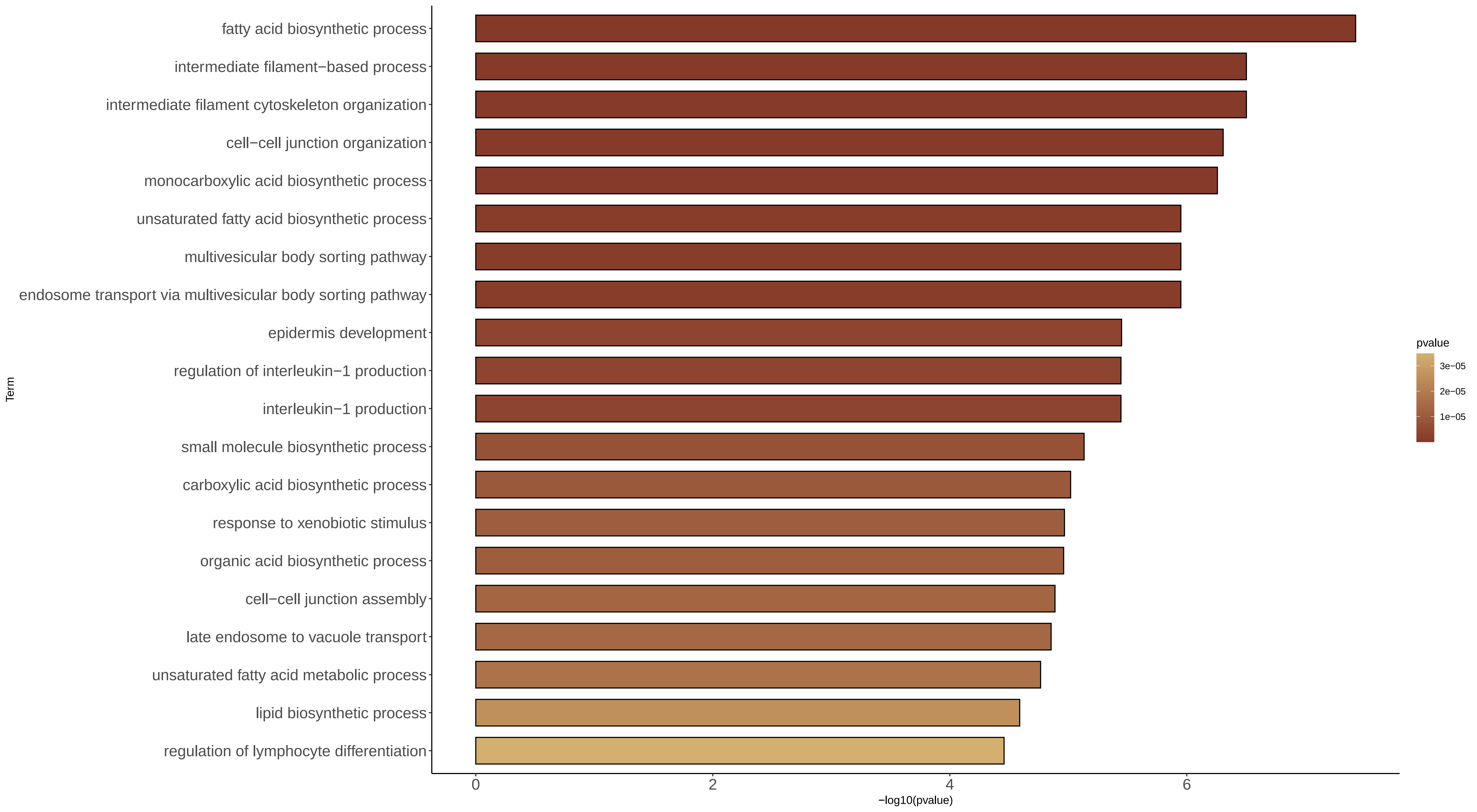
fatty acid biosynthetic process
intermediate filament−based process
intermediate filament cytoskeleton organization
cell−cell junction organization
monocarboxylic acid biosynthetic process
unsaturated fatty acid biosynthetic process
multivesicular body sorting pathway
endosome transport via multivesicular body sorting pathway
epidermis development
regulation of interleukin−1 production
interleukin−1 production
small molecule biosynthetic process
carboxylic acid biosynthetic process
response to xenobiotic stimulus
organic acid biosynthetic process
cell−cell junction assembly
late endosome to vacuole transport
unsaturated fatty acid metabolic process
lipid biosynthetic process
regulation of lymphocyte differentiation
Term
0
2
4
6
−log10(pvalue)
pvalue
3e−05
2e−05
1e−05
